# Awareness, knowledge, attitudes, and behaviors related to COVID-19 in Libya: a nation-wide online survey

**DOI:** 10.11604/pamj.2021.40.156.29455

**Published:** 2021-11-16

**Authors:** Alhadi Jahan, Marwa Mohamed, Eman Alabani, Aymaan Almaziq, Huda Elarriesh, Fatma Alagelli, Farag Alhadar, Khadejah Ben Tahir, Hazar Berrah, Mohammed Abudabbous, Wedad Hamouda, Najla Albahloul, Joma Elzoubia, Amal Dier

**Affiliations:** 1 Faculty of Health Sciences, University of Ottawa, Ottawa, Canada,; 2 Faculty of Dentistry, University of Benghazi, Benghazi, Libya,; 3 Faculty of Medicine, University of Misrata, Misrata, Libya,; 4 Faculty of Medicine, University of Tripoli, Tripoli, Libya,; 5 Faculty of Medicine, Almergeb University, Al-Khums, Libya,; 6 Faculty of Dentistry, University of Misrata, Misrata, Libya

**Keywords:** COVID-19, Libya, awareness, knowledge, attitude, behavior

## Abstract

**Introduction:**

the World Health Organization declared the COVID-19 outbreak to be a global pandemic in March 2020. However, the pandemic cannot be ended overnight and more social distancing and other self-care measures are needed to protect our communities. Therefore, people´s awareness, knowledge, attitudes, and appropriate behaviors are instrumental to containing the pandemic. The aim of this study was to determine COVID-19 awareness, knowledge, attitudes, and related behaviors in Libya.

**Methods:**

a cross-sectional online survey was conducted from October 10^th^ to November 10^th^, 2020 in 24 cities in Libya. The participants were non-medical professionals who were living in Libya for at least 2 years and were at least 18 years old.

**Results:**

a total of 1018 participants completed the questionnaire, with ages ranging from 18-74 years (Mean ± SD = 33.49±13.24); nearly two-thirds were < 40, and 68.2% were females. Almost half of the participants considered the potential threat of COVID-19 to be low, and one in five reported that they were “Not worried at all” about getting COVID-19. In multivariate analyses, participants who were 40-49 years old, had master´s degrees or higher, and worked in the private sector reflected high mean scores for both knowledge and attitudes, while those who lived in the Eastern or Southern regions had lower mean attitude scores.

**Conclusion:**

the low levels of awareness as well as the attitudes and behaviors among the public in Libya are worrisome. This study highlighted profound gaps that may put Libyan communities at high risk of a COVID-19 explosion. Therefore, immediate action is needed to address public awareness and attitudes and to improve COVID-19 related behaviors among the Libyan public.

## Introduction

The acute respiratory syndrome coronavirus 2 (SARS-CoV-2) was acknowledged as a pandemic by the World Health Organization (WHO) in March, 2020 [1]. At the time of this writing (January 26, 2021), there have been nearly 100 million (99,363,697) confirmed cases worldwide, including over 2 million (2,135,959) deaths, as reported by the WHO [2].With the continuous increase in reported cases worldwide, it is important for individuals to quickly interpret and absorb the rapidly changing messages from governments about public health and to take immediate action to minimize their risk of infection and the spread of the virus.

Nonetheless, this unprecedented global crisis has also been marked by miscommunication regarding the imminent threat of COVID-19, leading to public confusion and inaction. As of today, we are unaware of any available effective treatment for COVID-19 [3], and with regard to vaccines, although promising results have been published, their availability in unstable states like Libya is questionable. Therefore, self-care strategies are the only option available in Libya to prevent the spread of this deadly virus. Examples of appropriate self-care practices include physical distancing, personal hygiene, and hand-washing, among others [4].

Awareness, knowledge, attitudes, and practices people hold and adhere to towards the disease play an integral role in determining a society's readiness to accept health authorities´ measures and determining a path through the pandemic. Unfortunately, poor awareness and harmful practices can lead to severe consequences affecting the population's health. This was confirmed by a study conducted in Saudi Arabia [5] that showed that a lack of public awareness can hinder appropriate preventative practices. Similar findings were reported in the United States [6], China [7], Ghana [8], and Egypt [9]. Furthermore, poor awareness, lack of knowledge, negative attitudes, and practices like underestimation, stigmatization, and panic over new infectious diseases can aggravate this pandemic over time [7]. Thus, adequate awareness, knowledge, and good risk perceptions and preparedness to respond to COVID-19 among the general population is essential to contain the disease at the community level. In this study, we sought to conduct a nation-wide online survey to determine the current awareness of COVID-19 in Libya, perceptions of the seriousness of the threat, levels of knowledge about its transmission and symptoms, and attitudes and behaviors of the Libyan community with regard to this outbreak.

## Methods

**Study design and participants:** a cross-sectional online survey was used to gather details on the Libyan public´s awareness, knowledge, attitudes, and behaviors associated with the COVID-19 pandemic. In order to fulfill the objective of the study while restricting physical contact with the respondents and obtain their responses as quickly as possible during the outbreak in Libya, we used an online Google form. To be included, participants had to be Libyan citizens or foreigners who had lived in Libya for at least 2 years (i.e., before the beginning of the outbreak), be 18 years of age or older, and be able to read and understand the Arabic language. All questionnaires that did not meet the above criteria were excluded from the study. Additionally, we excluded all healthcare professionals (e.g. physicians, nurses, dentists, etc.), as we assumed that their awareness, knowledge, and behavior about COVID-19 would be higher than those of the general public.

**Sample size calculation:** the sample size was estimated by using Epi Info web-based software, version 3. Since the literature search did not identify similar studies conducted among the general public in Libya, we hypothesized the probability of awareness and knowledge about COVID-19 in the Libyan public as 50%, the power is 99%, precision of ±5%, with a design effect of 1.0, and the minimum required sample size was initially estimated to be 664 participants. Additionally, we added 10% to the sample size to account for possible non-responses. Therefore, the final minimum sample size was estimated to be 730 participants.

**Data collection tool:** the questionnaire was designed based on previous studies [6,10]. The study protocol was approved by the research ethics committees of Misrata University, the Misrata Medical Centre, and the National Cancer Institute (Misrata). All participants were asked to provide consent before proceeding to the questionnaire. The pre-final version of the questionnaire was tested on 31 volunteers in order to evaluate the readability and clarity of the questions and to estimate the time required to complete the questionnaire. Data obtained during this phase were not included in the data analysis. Based on the pilot testing, the questionnaire was determined to be reliable (Cronbach´s alpha = 0.89) and the questions were clear and easy to understand by different age groups. The questionnaire was written in the Arabic language and took about 7 minutes to complete.

The questionnaire consisted of four parts, as follows. The first part collected the sociodemographic information of the participants such as place of residence, gender, age, marital status, education, number of family members, occupation, and income (Table 1). The second part of the questionnaire entailed questions about the participants´ levels of awareness about COVID-19. The perceived awareness of COVID-19 (colloquially named “coronavirus”) was examined by first asking the question: “On a scale of 1 to 10, how serious of a public health threat do you think the COVID-19 is or might become?”, with 1 being “no threat at all” and 10 being “a very serious public health threat”. Furthermore, the participants were asked to rate their levels of worry about getting the coronavirus (very worried; somewhat worried; a little worried; not worried at all), while the last question was “Do you think that you will get sick from the coronavirus? (not at all; it´s possible; I definitely will). In this section, we also asked questions to check whether the participants currently had or had previously had the virus and whether they had been tested, were waiting for results, or had not had or were unable to have the test (Table 2 lists all the questions and possible responses). The third part of the questionnaire asked questions about the participants´ knowledge (18 items) and attitudes (14 items) towards COVID-19 (Table 3 lists all questions). Answers to these questions were “agree”, “disagree”, or “do not know”.

**Table 1:** sociodemographic characteristics (N=1018 participants)

Variables	Reference	Frequency	Percentage (%)
**Geographic location**	Western region	218	21.4
	Middle region	478	47
	Eastern region	185	18.2
	Southern region	137	13.5
**Gender**	Male	324	31.8
	Female	694	68.2
**Age**	18 - 29	542	53.2
	30 - 39	178	17.5
	40- 49	129	12.7
	50- 59	118	11.6
	60+	51	5
	Mean (SD)	33.49 (13.25)	
	Median (range)	28.00 (61)	
**Marital Status**	Single	520	51.1
	Married	456	44.8
	Divorced	14	1.4
	Widowed	28	2.8
**Education**	Secondary	413	40.6
	Bachelor	427	41.9
	Master or more	126	12.4
	No formal education	52	5.1
**Family members**	Less than 2	32	3.1
	2 - 4	285	28
	5- 7	401	39.4
	More than 7	300	29.5
**Occupation**	Student	345	33.9
	Public sector employee	358	35.2
	Private sector employee	142	13.9
	Unemployed, housewife	160	15.7
	Retired	13	1.3
**Income**	Less than 500	102	10
	500- 1000	392	38.5
	1000 - 2000	314	30.8
	2000- 5000	155	15.2
	More than 5000	55	5.4

**Table 2:** awareness of COVID-19 in overall sample (N=1018)

Questions	Mean (SD)		
Q1. On a scale of 1 to 10, how serious of a public health threat do you think the coronavirus is or might become? (1 being no threat at all, 10 being a very serious public health threat).	5.28 (2.76)		
**Questions**	**Responses**	**N**	**Percentage**
Q2. How worried are you about getting the coronavirus?	Not worried at all	188	18.5
	A little worried	367	36.1
	Somewhat worried	352	34.6
	Very worried	111	10.9
Q3. Do you think that you will get sick from the coronavirus?	Not at all	47	4.6
	It’s possible	863	84.8
	I definitely will	108	10.6
Q4. Do you know, or think, that you have (or previously had) the coronavirus?	Yes, previously had it	111	10.9
	Yes, currently have it	47	4.6
	No	693	68.1
Q5. Have you spoken (or did you speak) to a healthcare professional about this?	Yes	167	16.4
	No	781	76.7
	Don't know	26	2.6
Q6. Have you been tested for the coronavirus?	yes	331	32.5
	in process	14	1.4
	unable to get the test	1	0.1
	no	672	66
Q7. What was the outcome of this testing?	positive	163	16
	negative	177	17.4
	pending	10	1
	don't know	668	65.6
Q8. Do you know of anyone that has, or thinks they have the coronavirus, or previously had, or thought they had the coronavirus?	Yes, I know	807	79.3
	Yes, I think so	116	11.4
	Don't know	95	9.3

**Table 3:** knowledge of and attitudes toward COVID-19 pandemic in overall sample (N=1018)

Statements	Agree		Disagree		Don’t know	
	N	%	N	%	N	%
**Knowledge**						
Q1. The cause of COVID-19 is poor immunity?	812	79.8	124	12.2	82	8.1
Q2. The cause of COVID-19 is genetic disorders?	295	29	437	42.9	286	28.1
Q3. The cause of COVID-19 is transmission of the virus from infected person to healthy person?	954	93.7	20	2	44	4.3
Q4. The COVID-19 virus transmits through cough and sneeze?	964	94.7	23	2.3	31	3
Q5. The COVID-19 virus transmits through touching and hand shaking?	865	85	74	7.3	79	7.8
Q6. The COVID-19 virus transmits when using personal stuff of infected person?	881	86.5	58	5.7	79	7.8
Q7. Fever is a COVID-19 symptom	973	95.6	21	2.1	24	2.4
Q8. Cough is a COVID-19 symptom	883	86.7	58	5.7	77	7.6
Q9. Breathing difficulty is a COVID-19 symptom	965	94.8	15	1.5	38	3.7
Q10. Headache is a COVID-19 symptom	857	84.2	51	5	110	10.8
Q11. Sore throat is a COVID-19 symptom	779	76.5	93	9.1	146	14.3
Q12. Sweating is a COVID-19 symptom	520	51.1	173	17	325	31.9
Q13. Chest congestion is a COVID-19 symptom	524	51.5	182	17.9	312	30.6
Q14. Runny nose is a COVID-19 symptom	472	46.4	286	28.1	260	25.5
Q15. Tiredness is a COVID-19 symptom	879	86.3	76	7.5	63	6.2
Q16. Nausea or vomiting are COVID-19 symptoms	502	49.3	194	19.1	322	31.6
Q17. Sneezing is a COVID-19 symptom	652	64	192	18.9	174	17.1
Q18. Loss of taste and smell is a COVID-19 symptom	926	91	70	6.9	22	2.2
**Attitudes We can protect ourselves by**						
Q1. Staying away of sick people	940	92.3	68	6.7	10	1
Q2. Not touching the face by hands	918	90.2	60	5.9	40	3.9
Q3. Self-isolation when feeling sick	982	96.5	26	2.6	10	1
Q4. Using hand sanitizer	951	93.4	43	4.2	24	2.4
Q5. Washing hands very well	1004	98.6	10	1	4	0.4
Q6. Physical distancing	944	92.7	50	4.9	24	2.4
Q7. Staying at home	842	82.7	144	14.1	32	3.1
Q8. Wearing masks	967	95	38	3.7	13	1.3
Q9. Wearing gloves	638	62.7	289	28.4	91	8.9
Q10. Closing unnecessary stores (e.g., furniture, clothes etc.)	972	95.5	30	2.9	16	1.6
Q11. Closing schools and universities	900	88.4	72	7.1	46	4.5
Q12. Closing the borders with neighboring countries	930	91.4	41	4	47	4.6
Q13. Closing the airports	968	95.1	33	3.2	17	1.7
Q14. Limiting travel between cities	997	97.9	14	1.4	7	0.7

The last part of the questionnaire focused on collecting data about COVID-19-related behaviors. Participants were asked about any changes to their daily routines due to the COVID-19 pandemic, with possible answers being “no change at all”, “changed a little bit”, “somewhat changed”, or “changed a lot”. Participants also answered questions about how often they wear masks in public places (answers were “always”, “often”, “sometimes”, “rarely”, “never”, or “refuse to answer”; how comfortable were in doing some activities such as visiting friends, attending gatherings, or going to doctor´s appointments, with answers being “very comfortable”, “somewhat comfortable”, “not that comfortable”, “not comfortable at all”, or “don´t know”. They were also asked questions about their preparedness for another widespread coronavirus outbreak, with answers being “very prepared”, “somewhat prepared”, “a little prepared”, or “not prepared at all”, and about their confidence that the Libyan government could prevent further outbreaks of the coronavirus (answers were: “not confident at all”, “not very confident”, “somewhat confident”, or “very confident”). Table 4 lists all questions.

**Table 4:** COVID-19 related behaviors in the overall sample (N = 1018)

Questions	Responses	N	%
**Q1. Do you think that your daily routine has changed due to covid19?**	No change at all	108	10.6
	Changed a little bit	315	30.9
	Somewhat changed	364	35.8
	Changed a lot	231	22.7
**Q2. How often are you leaving your home?**	Once per day	375	36.8
	Multiple times per day	340	33.4
	once per week	128	12.6
	multiple times per week	135	13.3
	I never leave my home	40	3.9
**Q3. How often do you wear a mask when you are inside a store?**	Always	401	39.4
	Often	240	23.6
	Sometimes	170	16.7
	Rarely	92	9
	Never	106	10.4
	Refused to answer	9	0.9
**Q4. How comfortable are you now doing shopping in stores?**	Very comfortable	116	11.4
	Somewhat comfortable	432	42.4
	Not that comfortable	357	35.1
	Not comfortable at all	71	7
	Don’t know	42	4.1
**Q5. How comfortable are you now visiting your doctor in person?**	Very comfortable	180	17.7
	Somewhat comfortable	444	43.6
	Not that comfortable	252	24.8
	Not comfortable at all	55	5.4
	Don’t know	87	8.5
**Q6. How comfortable are you now inviting friends to your home?**	Very comfortable	177	17.4
	Somewhat comfortable	339	33.3
	Not that comfortable	268	26.3
	Not comfortable at all	145	14.2
	Don’t know	89	8.7
**Q7. How comfortable are you now attending a larger gathering of more than50 people?**	Very comfortable	56	5.5
	Somewhat comfortable	184	18.1
	Not that comfortable	361	35.5
	Not comfortable at all	341	33.5
	Don’t know	76	7.5
**Q8. How prepared do you think you are for another widespread coronavirus outbreak?**	Not prepared at all	199	19.5
	A little prepared	535	52.6
	Somewhat prepared	80	7.9
	Very prepared	204	20
**Q9. How confident are you that the Libyan government can prevent further outbreak of the coronavirus?**	Not confident at all	561	55.1
	Not very confident	346	34
	Somewhat confident	99	9.7
	Very confident	12	1.2

**Procedure**: the final version of the questionnaire was circulated through several online platforms, including social media and the official pages of community organizations, sports clubs, colleges and universities, and the official websites of 24 municipalities across the country representing the four geographical regions of Libya: the Western, Middle, Eastern, and Southern regions. The questionnaire was made accessible over a period of one month, from October 10 to November 10, 2020.

**Study variables**: the sociodemographic characteristics of the respondents such as place of residence, gender, age, marital status, education, number of family members, occupation, and income were considered as explanatory/independent variables. Other study variables (i.e., awareness, knowledge, attitude, and COVID-19 related behavior) were considered as response/dependent variables. The responses for knowledge and attitude were scored as 1 = correct answer, and 0 = wrong or do not know. The total score was the sum of all correct answers, which ranged from 0-18 (for knowledge) and 0-14 (for attitudes).

**Data analysis**: after checking for missing data, all completed questionnaires were imported, coded, and analyzed using the Statistical Package for Social Sciences (SPSS) version 26.0, and descriptive statistics were calculated for all the participants´ characteristics and survey responses. Dichotomous variables were expressed in frequency and percentage, while continuous variables were expressed as mean ± standard deviation. Bivariate and multivariate analyses were used as appropriate in order to test the significant associations between the participants´ characteristics and responses regarding COVID-19 awareness, knowledge, attitude, and behavior. An alpha level below 0.05 was considered for statistical significance (2-sided tests).

## Results

The questionnaire was accessed 1423 times, with 1018 completed questionnaires (response rate = 71.5%). In the following section, the respondents´ sociodemographic characteristics as well as the findings regarding the study variables, i.e., awareness, knowledge, attitudes, and COVID-19-related behaviors, are presented.

**Sociodemographic characteristics:** a total of 1018 participants completed the questionnaire, with the respondent´s ages ranging from 18-74 years (Mean ± SD = 33.49±13.24). The respondents were young overall; nearly two-thirds were > 40 years old, and 68.2% were female. The sample was representative of the four geographic regions of Libya, with nearly half of the respondents (47%) living in the middle region. Nearly half of the participants were socioeconomically disadvantaged, with monthly incomes of less than 2,000 Libyan dinars (LD). More than half the respondents had at least a bachelor´s degree, and a little more than one-third (39.4%) were living with at least five family members (Table 1).

**COVID-19 awareness:** overall, the participants´ awareness about COVID-19 was moderate, with a little over half of the participants considering the potential threat to be high (Table 2). Almost one in five (18.5%) reported that they were “Not worried at all” about getting COVID-19, and only 10.9% said they were “very worried” about getting the virus. Out of the 1018 participants, only 15.5% currently had the virus or had had it in the past. The vast majority of the respondents (84.8%) said that it was possible to get sick from the COVID-19 virus.

**COVID-19 knowledge:** most of the respondents were knowledgeable about the mode of transmission and the common symptoms of COVID-19. Regarding mode of transmission, the majority of respondents agreed that COVID-19 was transmitted from an infected to healthy person (93.7%), through coughing or sneezing (94.7%), and when using an infected person personal item (86.5%). In terms of COVID-19 symptoms, almost all the respondents agreed that fever (95.6%), cough (86.7%), and breathing difficulty (94.8%) were among the common symptoms of COVID-19 infection (Table 3).

**Attitude:** overall, the respondents showed a good attitude regarding the protective measures suggested by the WHO to contain the spread of COVID-19, and agreed that wearing masks, physical distancing, and hand-washing were among the protective measures against COVID-19, at 95%, 92.7%, and 98.6% respectively. Similar responses (around 95.5%), were reported regarding closing unnecessary stores (e.g., furniture and clothing stores, etc.), closing the borders (91.4%), and closing the airports (95.1%) to control the spread of COVID-19. In terms of school and university closures, about 88.4% of the respondents agreed that this was necessary to contain the pandemic (Table 3).

**COVID-19-related behaviors:** about one in five of the respondents (22.7%) reported that the COVID-19 pandemic had changed their daily routine a lot. A total of 36.8% of the study sample reported that they would leave their house only once a day, whereas 33.4% reported that they would leave their home multiple times a day. In contrast, only 3.9% said that they never left their homes. We also asked questions to investigate the public´s level of preparedness for other outbreaks and their trust in the current government to prevent them. One in five respondents (19.5%) reported that they were “not prepared at all”, and more than half the respondents (52.6%) reported that they were “a little prepared” for another wave of COVID-19. The majority of respondents (89.1%) were “not confident at all” or “not very confident” that the Libyan government could prevent further outbreaks of the COVID-19 virus (Table 4).

**Regression analysis:** we performed multiple regression analyses to check for any associations between the levels of awareness, attitude, and reported actions and sociodemographic variables such as geographic location, gender, age, marital status, education, number of family members, occupation, and monthly income. Age, education, and occupation were significantly associated with the level of knowledge related to COVID-19 symptoms, with p-values of 0.018, 0.000, and 0.003 respectively. The highest mean score for respondents´ knowledge was 9.55 ± 2.79 for the age group 40-49 years. For the education variable, the highest mean score for knowledge about symptoms was reported by respondents who had master´s degrees or higher, at 10.2 ± 2.47. In terms of occupation, it turned out that private sector employees reported the highest mean score for knowledge about symptoms, at 10.5 ± 2.26. Correlation between attitudes was significant, with geographic location, education, and occupation reflecting p-values of 0.001, 0.005, and 0.000 respectively (Table 5).

**Table 5:** levels of knowledge and attitude as per sociodemographic variables which found significant (N = 1018)

Variable	N	Mean score	Standard Deviation	P-Value
			**Knowledge**	
**Age groups**				**0.018**
18-29	542	8.63	2.35	
30-39	178	8.85	2.51	
40-49	129	9.55	2.79	
5-59	118	9.07	2.72	
60+	51	8.71	2.75	
**Education**				**0.000**
Secondary	413	8.64	2.58	
Bachelor	427	8.78	2.35	
Master or more	126	10.2	2.47	
No formal education	52	8.62	2.67	
**Occupation**				**0.003**
Student	354	9.09	2.64	
Public sector	358	8.81	2.56	
Private sector	142	10.5	2.26	
Unemployed	160	8.7	2.34	
Retired	13	8.35	2.55	
			**Attitude**	
**Geographic location**				**0.001**
Western region	218	13	1.54	
Middle region	478	13	1.73	
Eastern region	185	12.8	1.62	
Southern region	137	12.5	2.06	
**Education**				**0.005**
Secondary	413	12.6	1.85	
Bachelor	427	12.8	1.78	
Master or more	126	13.2	1.88	
No formal education	52	12.6	2.05	
**Occupation**				**0.000**
Student	354	12.4	2.03	
Public sector	358	12.6	2.07	
Private sector	142	13.5	0.66	
Unemployed	160	13.1	1.54	
Retired	13	12.8	1.64	

## Discussion

This cross-sectional study was aimed at providing evidence about the awareness, knowledge, attitudes, and behaviors towards COVID-19 among Libyans. This was the first nation-wide survey to represent the four geographic regions of Libya: Western, Middle, Eastern, and Southern. Notably, the majority of the study participants were females (68.2%), which might be due to gender differences in responding to online surveys, as reported in previous studies [11]. A little more than half the respondents (53.2%) corresponded to the age group 18-29 years, which represented the actual demographic figures in Libya [12]. To maintain the quality of our data, we purposively excluded healthcare professionals, as we assumed that they had sufficient awareness, knowledge, attitudes, and appropriate behaviors regarding the COVID-19 pandemic.

Overall, the findings show that public awareness about COVID-19 in Libya is moderate, as the majority of respondents perceived the threat of a COVID-19 outbreak to be moderately serious on a scale of 1 to 10 (Mean score ± SD = 5.28±2.76). A study carried out by Wolf *et al*. [6]in the United States used the same scale, and found that most Americans perceived COVID-19 as serious health threat (Mean score ± SD = 9.0±1.7). A more comparable study was conducted in Egypt by Abdelhafiz *et al*. [9], who found that the majority of respondents (86.9%) identified COVID-19 as a high risk threat to human health. This variability in the public perception of the seriousness of the COVID-19 pandemic in Libya compared to other countries may reflect the value of educational programs in those countries that are lacking in Libya.

However, our respondents showed high levels of knowledge regarding the sources of infection and symptoms of COVID-19. For example, 94.7% of the respondents knew that the COVID-19 virus can be transmitted through coughing and sneezing, 85% knew that the COVID-19 virus can be transmitted through touching and hand-shaking, and 86.5% knew that the COVID-19 virus can be transmitted when using an infected person´s personal items. A study done in Ghana by Serwaa *et al*. [8] found that the overall knowledge about the COVID-19 among Ghanaians was 61.7%, and similarly, a study carried out in the United Arab Emirates by Bhagavathula *et al*. [13] reported respondents´ poor knowledge of COVID-19 transmission and symptoms, with overall percentages of 61% and 63.6%, respectively.

The high knowledge percentages reported in our study may, however, be due to the timing of our survey, since the above-mentioned studies were conducted at the beginning of the COVID-19 outbreak, while our study was conducted in October (i.e., 7 months after the WHO declared COVID-19 to be a global pandemic). Therefore, people in Libya had a better chance to learn more about the virus and gain more knowledge about its transmission and the common symptoms of the infection. In this study, regression analyses were also conducted in order to examine whether public knowledge and attitudes varied based on sociodemographic characteristics. In our population, we found a significant positive correlation between COVID-19-related knowledge and the variables for age, education, and occupation (Table 5). Respondents who were between 40-49 years old showed the highest mean score (out of 18 total score) for the knowledge variable (Mean ± SD = 9.55±2.79, P-value =0.018), and we also found a significant correlation between knowledge and education, where respondents who had a masters´ degree or higher reported the highest mean score, with (Mean ± SD= 10.2 ± 2.47, p-value > 0.001). This finding is not surprising, because it has been documented in earlier studies that individuals with higher education report more knowledge compared with other categories [10]. However, what is surprising is that private sector employees reported the highest mean scores for knowledge (Mean ± SD= 10.5 ± 2.26, p-value = 0.003) compared to public sector employees and unemployed respondents. This finding may reflect the significance of employee education initiatives that have been implemented recently in several private sector facilities. This also highlights the urgent need for similar programs in the public sector to improve the overall knowledge in the community and consequently help in pandemic control in the country at large.

In terms of attitudes towards the COVID-19 pandemic, we found that the mean attitude score was significantly correlated with geographic location, education, and occupation. People who lived in the Middle and Western regions showed slightly higher attitude mean scores (Mean ± SD= 13 ± 173, p-value = 0.001) than those who lived in the Eastern and Southern regions. In Libya, most of the Southern region, for instance, comprises rural areas (mostly in the Sahara) that lack appropriate infrastructure to keep track of the continuous updates about the pandemic. This poor attitude may also be connected to the cultural and ethnic diversity in those regions, which needs further attention. The findings of this study are of utmost importance, however, since they may provide data on implementing programs that target people who might be unable to access useful educational platforms due to financial or technical reasons.

It is clear that the evolving COVID-19 outbreak requires social distancing and other measures to protect public health; however, recommendations in Libya have been inconsistent, unclear, and often confusing to the public. This was confirmed by our study, as only a little over half of the participants considered the potential threat of COVID-19 to be high (Table 2). Moreover, the vast majority of the respondents seemed “don´t care” about getting the infection or spreading it to others, and about one in five (18.5%) reported that they were “Not worried at all” about getting COVID-19. This finding is alarming because misinformation in relation to COVID-19 can be a serious threat to all of humanity in the social media era, as reported by several studies [14,15]. Exposure to high volumes of fake news and misleading information, especially on social media, can cause serious outcomes, such as the refusal and denial of essential health behaviors. Furthermore, misinformation and rumours can push the public to respond against scientifically proven recommendations [16]. In Nigeria for instance, public health authorities reported several cases of poisoning with chloroquine, an anti-malaria medication, after the spread of fake news about its effectiveness in treating COVID-19 [17]. A more severe influence of misleading information was reported in India, where a 50-year-old father of three children committed suicide when he was informed about getting a viral infection which was wrongly connected to COVID-19 [18]. The situation in Libya, as evidenced by our findings, is concerning, as social media is open to everyone without any legal restrictions due to the fragile political and economic state of the country.

At the individual level, several self-care practices have been recommended by the WHO to control for the spread of COVID-19 in the community. For instance, wearing masks, washing hands, physical distancing, and self-isolation when feeling unwell are among the common practices that have been in place and have been supported by the literature. In our study, the majority of respondents agreed that wearing masks, for instance, is a good strategy to combat the virus; however, only 39.4% of them said they wore masks in stores, and one in ten said that they never wore them at all. This finding is disturbing, since wearing masks in public places like grocery stores is among the best possible strategies to control the pandemic [19]. Findings similar to ours were reflected in a study in Egypt [9], where researchers reported that only 35% of their study participants were willing to wear masks in public places. Poor adherence to self-care practices puts the public at high risk of spreading the virus. This negative attitude of the Libyan public can be attributed to the loose government policies in this regard, and we recommend that government and local authorities make strict rules to force the public to wear masks in public places in order to help contain the spread of COVID-19.

In Libya, the situation has been exacerbated by the intermittent military conflicts in many parts of the country for nearly 10 years. In June 2020, the WHO urged all armed groups in Libya to enable humanitarian organizations to access healthcare facilities, especially in the south, to help combat the increase in the country's COVID-19 cases. As of December 2019, more than 300,000 people had been displaced because of the war [20], with most of them living in poor conditions that allow the virus to spread easily. Moreover, the country´s healthcare system is struggling, with most primary health care institutions at higher risk of closures due to shortages of medical staff and supplies, including medications and equipment. As highlighted by Daw *et al*. [21], the healthcare system in Libya is malfunctioning due to the political instability and the lack of a central government to control healthcare delivery. According to the WHO´s Joint External Evaluation, which classifies countries according to their preparedness to find, stop, and prevent epidemics, Libya falls under the “not ready” category [22]. Therefore, implementing self-care strategies and improving public awareness and preparedness is urgently needed.

A few limitations of this study should be noted. First, the data used for this study were collected solely online, which might have recruited only participants who are more interested or concerned about COVID-19 than the general public. Second, the number of female participants was almost twice that of their male counterparts. In fact, this is a shared limitation among many studies that have used the same methodology [10,23], which has been connected with gender differences between males and females in responding to online surveys. Lastly, the collected data may be biased by the respondents´ honesty and ability to recall information, which may influence the generalizability of the findings. Despite these limitations, however, our study is the first nation-wide survey to provide valuable data about awareness, knowledge, attitude, and behaviors related to the COVID-19 pandemic in Libya.

## Conclusion

Almost a year has passed since the first case of COVID-19 was announced in Wuhan, China; however, the awareness, knowledge, and behaviors among the general public in Libya are worrisome. Our study highlights profound gaps that may put Libyan communities at high risk of a COVID-19 explosion. The COVID-19 pandemic has the potential to put more pressure on the exhausted healthcare system in Libya due to poor awareness and inadequate preventative behaviors among the public. Our study also provides evidence on those who are socioeconomically disadvantaged and live with limited resources. Immediate actions are needed now to protect Libya from this deadly virus, and the first step toward containing the pandemic is by addressing awareness, knowledge, and research-based behaviors among the public, especially in vulnerable populations. As the COVID-19 graph for Libya is ascending as more cases are confirmed every day and the second wave of the virus is near, with new traits being confirmed in many parts of the world, it is imperative to take tangible steps at personal and community levels to increase the awareness, knowledge, and appropriate behaviors to fight the pandemic.

### What is known about this topic

- There is miscommunication regarding the imminent threat of COVID-19, leading to public confusion and inaction;
- Self-care strategies are the only option available now to prevent the spread of this deadly virus;
- Poor awareness, knowledge, and harmful practices regarding COVID-19 can lead to severe consequences affecting population health.

### What this study adds

- Libyan people´s awareness about COVID-19 is moderate;
- Respondents agreed that wearing masks, physical distancing and hand-washing were protective measures against COVID-19;
- Individuals who live in rural areas have lower attitude scores overall regarding the COVID-19 pandemic.
